# 

**Published:** 1892-09-10

**Authors:** 


					price twopence.
Jll, Vol. XII.?Sept. 10th, 1892.
^Jtered at the General Post Office ai a Newspaper for
^omission in the United Kingdom and Abroad.]
t H
WITH EXTRA NURSlNU aUKh-LtivitiN r
Per Annum, 8s. 6d., post free.
Monthly Farts, 6d.; post free, 8d.
Ditto per Annum, 8s.t post free.
No.
311. Vol. XII. J Saturday,
September 10th, 1892.
TTwopenoe.

				

